# A cohort study of the risk of cancer associated with type 2 diabetes

**DOI:** 10.1038/sj.bjc.6605240

**Published:** 2009-08-18

**Authors:** A A Ogunleye, S A Ogston, A D Morris, J M M Evans

**Affiliations:** 1 Section of Public Health, Division of Clinical and Population Sciences and Education, University of Dundee, Mackenzie Building, Kirsty Semple Way, Dundee DD2 4BF, Scotland, UK; 2 Diabetes Research Centre, University of Dundee, Ninewells Hospital, Dundee DD1 9SY, Scotland, UK

**Keywords:** type 2 diabetes, cohort study, survival analysis, epidemiology

## Abstract

**Background:**

There is evidence to suggest that diabetes may increase the risk of incidence and mortality from cancer.

**Methods:**

In a cohort study using record-linkage health-care datasets for Tayside, Scotland in 1993–2004, we followed up 9577 newly diagnosed patients with type 2 diabetes, and two matched non-diabetic comparators, in the national cancer register.

**Results and conclusions:**

The risk ratio for any cancer, adjusted for deprivation, was 0.99 (95%CI 0.90–1.09). Significantly increased risks were observed for pancreatic, liver and colon cancer.

Whether there was an increased prevalence of cancer associated with insulin use was questioned in the early twentieth century (Marble, 1934), and there is a growing body of evidence suggesting that diabetes increases the risk of the incidence and mortality from certain cancers. These include pancreatic and liver cancer, colorectal cancer, breast cancer, bladder cancer, cancer of the kidney and endometrial cancer (Wideroff *et al*, 1997; Coughlin *et al*, 2004; Rousseau *et al*, 2006; Kuriki *et al*, 2007). However, the results of studies of diabetes and cancer have been very mixed with many not distinguishing between type 1 and type 2 diabetes (Wideroff *et al*, 1997), or have defined groups according to treatment (Swerdlow *et al*, 2005). In some studies, the basis for diabetes diagnosis is self-report (Coughlin *et al*, 2004), in others it is clinical (Saydah *et al*, 2003). Studies have been both retrospective (Kuriki *et al*, 2007) and prospective, with some prospective studies following up from diagnosis of diabetes (Ragozzino *et al*, 1982); another followed up a cross-sectional sample of patients (Swerdlow *et al*, 2005). In this study, we present the risks of different cancers following diagnosis of type 2 diabetes in Tayside, Scotland, using a standardised methodology.

## Materials and methods

This cohort study was carried out using the datasets of the Health Informatics Centre (HIC), University of Dundee, which developed the record linkage of routinely collected health-care datasets to facilitate epidemiological and other health research in the population of Tayside Health Board, Scotland (approximately population 400 000) (Evans *et al*, 1995). A diabetes clinical information system was used to identify all patients registered with any Tayside GP practice who were diagnosed with type 2 diabetes in 1993–2004 (Morris *et al*, 1997). Their date of diagnosis was defined as their study index date. Any patient who had an earlier record of cancer diagnosis on the Scottish national cancer registry (SMR6) was excluded (Scottish Cancer Registry, 2009).

For each eligible patient with type 2 diabetes, two non-diabetic comparators were selected at random from computerised lists of patients registered with primary care, matched for age (within 1 year), sex and GP practice. The index date of the comparator was that of its matched diabetic patient; and the comparator had to be alive and have no earlier cancer diagnosis on this date.

Diabetic patients and their comparators were followed for a maximum of 11 years in a survival analysis to the study end date (1st January 2004). The primary outcome was diagnosis of malignant cancer on SMR6; deaths were also identified. The relationship between type 2 diabetes and cancer was assessed in a Cox regression unadjusted, and then in a multivariable model adjusted for deprivation (measured using deciles of a postcode score for material deprivation) (Carstairs, 1990). This was repeated for specific cancer types according to the ICD10 diagnosis code recorded on SMR6.

Statistical analyses were performed using SPSS (Statistical Package for Social Scientist) 15.0 software programme. All data were anonymised for analysis. Ethical approval was obtained from the Multi-Centre Research and Ethics Committee for Scotland.

## Results

The study population comprised 9577 people registered with any Tayside general practice diagnosed with type 2 diabetes between 1993 and 2004, and 19 154 non-diabetic comparators, of these, 53.3% were male, and the mean age at index date was 62 years; 661 (6.9%) of patients with diabetes were diagnosed with cancer, as recorded on SMR6, during follow-up (mean length of follow-up 1417 days), compared with 1364 (7.1%) of the comparators (mean follow-up 1476 days). Of the diabetic patients, 12.5% died and 8.3% of the comparators died.

The unadjusted risk ratio for any diagnosis of cancer was 1.01 (95% CI 0.92–1.11) for diabetic patients. After adjusting for deprivation, it reduced to 0.99 (95% CI 0.90–1.09) (Table 1). Risk ratios were determined for specific cancers, presented in Table 1 in decreasing order of frequency (accounting for 79% of all cancers). There were significant increased risks associated with type 2 diabetes and of colon, pancreatic and cancer liver only. The results were largely unchanged after excluding outcomes in the first year of follow-up.

## Discussion

This study finds no evidence that patients with type 2 diabetes have an overall increased risk of cancer compared with non-diabetic people, with adjusted risk ratios approaching unity. However, there was an increased risk of certain specific cancers that, because uncommon, did not have a substantial effect on the overall risk ratio.

Patients with type 2 diabetes were three times more likely to develop pancreatic cancer than non-diabetic people, a widely reported association (Coughlin *et al*, 2004; Rousseau *et al*, 2006; Kuriki *et al*, 2007). Although type 2 diabetes could be a risk factor for pancreatic cancer (Wideroff *et al*, 1997), it is more likely to be a consequence (reverse causality) (Gullo *et al*, 1994), being stronger among recently diagnosed patients. The three-fold increased risk of liver cancer is also similar to earlier reports (Wideroff *et al*, 1997; Rousseau *et al*, 2006), some of which have adjusted for confounding by alcohol and viral hepatitis (Davila *et al*, 2005). However, the risk is higher after excluding patients diagnosed in the year following diabetes, suggesting that reverse causality is unlikely to be the explanation here.

Our slight increased risk of 1.46 for cancer of the colon is in line with a recent meta-analysis (Larsson *et al*, 2005). Other studies have observed similar risks, some of which were not significant perhaps owing to small sample size (Wideroff *et al*, 1997; Coughlin *et al*, 2004; Rousseau *et al*, 2006). In contrast, we observed no increased risk for rectal cancer, consistent with the lack of statistically significant increased risk in the Nurses' Health Study (Hu *et al*, 1999).

This study provided no evidence for associations between type 2 diabetes and other cancer types. A Danish study found increased risks of kidney and endometrial cancer (Wideroff *et al*, 1997), these cancers were not common in our cohort and larger sample sizes would be needed to detect an associated risk. We also found no increase of breast or bladder cancer, despite a US finding of excess mortality (Coughlin *et al*, 2004). However, associations with mortality could be confounded by survival-related factors.

We are confident in the validity of our data sources, which have been widely used in epidemiological studies, whereas SMR6 is a national, validated dataset. It also allowed estimation of risks of cancer incidence, using precise dates of diagnosis. The study's main limitation was lack of information on lifestyle-related confounders, although we were able to adjust for deprivation. Patients with diabetes may have a higher prevalence of lifestyle-related risk factors for cancer, such as obesity (Hjartaker *et al*, 2008). However, we found no increased risks of many cancers despite these potential confounders, thereby increasing confidence in the conclusions.

## Figures and Tables

**Table 1:** Unadjusted and adjusted risk ratios for any malignant cancer and specific cancers among patients with type 2 diabetic people and their comparators in Tayside, Scotland, 1993–2004

	**No (%) of cancer (diabetes)**	**No (%) of cancer (comparators)**	**Unadjusted RR (95% CI)**	**Adjusted RR (95% CI**)^a^	**Adjusted RR (95% CI) (excluding outcomes in 1st year)**
Any malignant cancer C00–97	661 (6.9%)	1364 (7.1%)	1.01 (0.92–1.11)	0.99 (0.90–1.09)	1.05 (0.93–1.18)
Skin C43–44	128 (1.3%)	322 (1.7%)	0.83 (0.68–1.02)	0.84 (0.68–1.03)	0.83 (0.64–1.07)
Lung C33–34	77 (0.8%)	198 (1.0%)	0.81 (0.62–1.05)	0.77 (0.59–1.01)	0.70 (0.49–0.99)
Colon C18–19	67 (0.7%)	95 (0.5%)	1.49 (1.09–2.03)	1.46 (1.07–2.01)	1.56 (1.05–2.32)
Breast C50	52 (0.5%)	103 (0.5%)	1.05 (0.76–1.47)	1.05 (0.75–1.47)	1.05 (0.71–1.57)
Prostate C61	43 (0.4%)	117 (0.6%)	0.77 (0.54–1.09)	0.77 (0.54–1.09)	0.76 (0.50–1.17)
Pancreas C25	31 (0.3%)	20 (0.1%)	3.19 (1.82–5.60)	3.06 (1.73–5.39)	2.85 (1.27–6.43)
Oesophagus C15	24 (0.3%)	28 (0.1%)	1.79 (1.04–3.08)	1.70 (0.98–2.95)	1.74 (0.91–3.32)
Bladder C67	17 (0.2%)	51 (0.3%)	0.69 (0.40–1.20)	0.70 (0.40–1.21)	0.53 (0.24–1.15)
Non-Hodgkins C82–85	17 (0.2%)	26 (0.1%)	1.37 (0.74–2.51)	1.36 (0.74–2.51)	1.44 (0.67–3.10)
Liver C22	17 (0.2%)	12 (0.1%)	2.96 (1.41–6.19)	2.93 (1.40–6.14)	3.50 (1.38–8.91)
Stomach C16	16 (0.2%)	46 (0.2%)	0.73 (0.41–1.29)	0.73 (0.41–1.29)	0.77 (0.36–1.66)
Rectum C20	13 (0.1%)	48 (0.3%)	0.57 (0.31–1.06)	0.56 (0.30–1.03)	0.53 (0.25–1.10)
Endometrium C54	13 (0.1%)	17 (0.1%)	1.60 (0.78–3.29)	1.47 (0.70–3.09)	0.94 (0.33–2.70)
Kidney C64	6 (0.1%)	24 (0.1%)	0.52 (0.21–1.28)	0.52 (0.21–1.27)	0.25 (0.06–1.06)

Abbreviations: CI=confidence intervals; RR=risk ratio.

a Adjusted for deprivation decile.
